# Teaming Up for Trouble: Cancer Cells, Transforming Growth Factor-β1 Signaling and the Epigenetic Corruption of Stromal Naïve Fibroblasts

**DOI:** 10.3390/cancers10030061

**Published:** 2018-02-27

**Authors:** Sergio Lamprecht, Ina Sigal-Batikoff, Shraga Shany, Naim Abu-Freha, Eduard Ling, George J. Delinasios, Keren Moyal-Atias, John G. Delinasios, Alexander Fich

**Affiliations:** 1 Department of Clinical Biochemistry and Pharmacology, Ben Gurion University of the Negev, Beersheva 8410500, Israel; sigalina@gmail.com (I.S.-B.); shany@bgu.ac.il (S.S.); 2 Faculty of Health Sciences, Ben Gurion University of the Negev, Beersheva 8410500, Israel; naimaf@clalit.org.il (N.A.-F.); ling@bgu.ac.il (E.L.); atiask@post.bgu.ac.il (K.M.-A.); fich@bgu.ac.il (A.F.); 3 Institute of Gastroenterology and Hepatology, Soroka University Medical Center, Beersheva 8410100, Israel; 4 Pediatrics Department B, Soroka University Medical Center, Beersheva 8410100, Israel; 5 International Institute of Anticancer Research, Kapandriti, Athens 19014, Greece; editor@iiar-anticancer.org (G.J.D.); iiar@iiar-anticancer.org (J.G.D.)

**Keywords:** cancer, tumor microenvironment, transforming growth factor-β, epigenetics, colorectal cancer, cancer-associated fibroblasts

## Abstract

It is well recognized that cancer cells subvert the phenotype of stromal naïve fibroblasts and instruct the neighboring cells to sustain their growth agenda. The mechanisms underpinning the switch of fibroblasts to cancer-associated fibroblasts (CAFs) are the focus of intense investigation. One of the most significant hallmarks of the biological identity of CAFs is that their tumor-promoting phenotype is stably maintained during in vitro and ex vivo propagation without the continual interaction with the adjacent cancer cells. In this review, we discuss robust evidence showing that the master cytokine Transforming Growth Factor-β1 (TGFβ-1) is a prime mover in reshaping, via epigenetic switches, the phenotype of stromal fibroblasts to a durable state. We also examine, in detail, the pervasive involvement of TGFβ-1 signaling from both cancer cells and CAFs in fostering cancer development, taking colorectal cancer (CRC) as a paradigm of human neoplasia. Finally, we review the stroma-centric anticancer therapeutic approach focused on CAFs—the most abundant cell population of the tumor microenvironment (TME)—as target cells.

## 1. Introduction

The reductionist viewpoint of neoplasia based on the autonomous behavior of tumor cells has been superseded by the mounting evidence that cancer is a complex micro-ecosystem comprising not only the transformed cells but also heterotypic non-cancer cell populations resident in the tumor microenvironment (TME), [1,2]. This view is providing new opportunities for therapeutic approaches [1,2,3]. Previously, stromal fibroblasts enclosing a growing tumor were not at the center of attention as key participants in the process of carcinogenesis. A vast number of studies have discounted this view and showed that the naïve fibroblasts are educated by the adjacent tumor cells to foster their growth program. The “activated” fibroblasts, referred to as myofibroblasts and in the cancer context “cancer-associated fibroblasts” (CAFs), are the most abundant cell population resident in the TME producing an array of oncogenic cytokines and chemokines [4,5,6,7,8,9].

Several cell types have been qualified as progenitors that acquire the CAF phenotype by trans-differentiation explaining, at least in part, the heterogeneity of CAFs in the TME [4,5,6,7,8,9]. Apart from normal resident fibroblasts, the list includes pericytes, adipocytes, smooth muscle cells, hepatic and pancreatic stellate cells to name but a few. Epithelial cancer cells are also able to acquire a CAF-like mesenchymal phenotype via the epithelial–mesenchymal transition (EMT), a trans-differentiation program that strongly favors migration and invasion of cancer cells [10]. Irrespective of their disparate cellular origin and lineage, the ultimate purpose of these progenitoronce morphed into CAFss to sustain the demanding lifestyle of the cancer cell. A note of caution is warranted at this point. Tumor stroma does not invariably act as a partner in crime but may restrain cancer growth, an inhibitory role shown in murine pancreatic cancer [11,12]. In this context, the secretion by CAFs of pro-collagen fibrils into the extracellular matrix (ECM)—referred to as desmoplasia—serves as a barrier impeding the growth of cancer cells instead of enhancing their biological aggressiveness. This protective stromal function proposed by J. Delinasios in early works [13] has been extensively reviewed [14,15].

An extensive survey of how cancer cells impose on non-transformed stromal fibroblasts—a pro-tumorigenic drive to foster their growth agenda—is not the aim of this review and readers seeking detailed information on this rich area of ongoing investigation are directed to several comprehensive reviews (refs. [4,5,6,7,8,9] and references therein). In the present review, among the molecular agents promoting the generation of CAFs, we have singled out TGF-β1, a master cytokine overexpressed by both tumor cells and stromal CAFs essential for the fibroblast-to-myofibroblast trans-differentiation and, consequently, for the shaping of the CAF phenotype [16,17,18,19]. TGF-β1 serves as a major conduit for communication between cancer cells and stromal myofibroblasts.

One of the most significant hallmarks of the biological identity of CAFs is that their tumor-promoting phenotype remains stable during in vitro and ex vivo propagation without the continual interaction with neighboring cancer cells. This intriguing finding, discussed in detail below, has been reported in a large number of studies [20,21,22,23]; see also citations in the following sections of this review.

Herein, for scrutiny and discussion, we have selected the aforementioned aspects of CAF biology which are of mounting interest for basic research and in translational medicine, and asked the following: *Which genomic changes underpin the long-term memory of co-evolution of CAFs with their rogue cancer neighbors, leading to the durability of the CAF phenotype? How does TGF-β1 signaling program the durable CAF phenotype? How the biological changes imposed on stromal fibroblasts by tumor cells and sustained by TGF-β1 affect cancer medicine?*

## 2. The Durable Gene Signature of Cancer-Associated Fibroblasts: Clonal Somatic Mutations or Epigenetic Changes?

The acquisition of the CAF phenotype is associated with the differential expression of a large number of genes compared to normal stromal fibroblasts. The altered gene signature—part and parcel of the pro-tumorigenic competence of CAFs—is read to assess prognostic relevance in cancer diseases and as a reference background to interrogate the molecular modes of action of specific agents during fibroblast-to-myofibroblast trans-differentiation [24,25,26].

As mentioned previously, the CAF phenotype is stable. A tenable interpretation of this functional independence from their neighbors is that somatic gene mutations in CAFs maintain their autonomous, pro-tumorigenic action. Indeed, studies reported loss of heterozygosity (LOH) and mutations in *TP53* and *PTEN* genes in stromal fibroblasts and in peritumoral mesenchymal cells adjacent to breast carcinomas [27,28,29,30,31]. LOH and TP53 mutations were also observed in human colorectal cancer (CRC) stromal fibroblasts [29]. Frequent gene dosage alterations in peritumoral stromal mesenchymal cells were detected in epithelial ovarian carcinomas [32]; see also the table in [33].

If this is so, a question arises: why do clones generating fibrosarcoma—such as tumors—not emerge from the abundant CAF population? Indeed, germline loss of a tumor suppressor gene function permanently alters the biological identity of stromal fibroblasts, and these mutant cells express frank hallmarks of transformation. Thus, colon stromal fibroblasts in Familial Adenomatous Polyposis, an inherited disease in which numerous adenomatous polyps form predominantly in the epithelium of the large intestine, were shown to immortalize [34]. Moreover, dermal fibroblasts isolated from patients with Li-Fraumeni syndrome, a hereditary disease frequently linked to germline mutations in the *TP53* gene, exhibit chromosomal aberrations, such as aneuploidy, and immortalize [35,36]—all hallmarks of cell transformation not found in CAFs.

Recently, Ezold and colleagues [37] examined primary dermal fibroblasts from monozygotic twin sisters discordant for childhood cancer with one sibling suffering from recurrent breast cancer due to a mosaic epi-mutation in the *BBCA1* gene. Transcriptome assays of *BBCA1* epi-mutant skin fibroblasts showed genetic changes typical of CAFs, while in the healthy twin sister skin fibroblasts were normal.

Cumulatively, these studies indicate that human germline mutations profoundly alter the phenotype of normal stromal fibroblasts: As mentioned above, these permanent biological changes are typically absent from stromal fibroblasts surrounding cancers that do not arise from inherited gene changes, collectively referred to as “sporadic” cancers. With this background in mind, and in sharp contrast with the studies proposing mutant genes as the origin of a stable CAF phenotype, a vast number of studies have shown that clonal somatic mutations are rarely detected in the tumor stroma. Thus, Corver et al. [38] reported that stromal cells in patients with cervical cancer were diploid and exhibited a phenotypic signature identical to that of patient-matched normal endometrium. In a carefully designed study, Allinen and colleagues [39] working with human breast carcinomas demonstrated that genetic alterations were present only in malignant epithelial cells and were absent from stromal cells. Consonant with these findings, Qiu and co-workers [40] in a genome-wide analysis noted that CAFs derived from human ovarian and breast cancers very rarely exhibit LOH and copy number alterations compared to tumor tissue specimens. Similar findings attesting to the genomic stability of CAFs were reported in a study of human ovarian cancer and of human breast carcinoma-associated fibroblasts [41,42]. Furthermore, analysis of genome-wide copy number changes and p53 immunohistochemical labeling of tissue microarrays in CAFs resected from human pancreatic cancer specimens failed to evidence somatic gene copy number losses or gain or *TP53* gene mutations [43].

A recent extensive study [44] has re-addressed in depth the issue of whether clonal gene aberrations are present in stromal cells contiguous to prostate carcinoma cells. Genomic DNA extracted from laser micro-dissected prostate cancer-associated stromal cells isolated from human fresh frozen prostate cancer tissue and from cultured prostate CAFs was examined using a wide range of methods, including array comparative genomic hybridization (CGH), DNA sequencing and microsatellite assays. In contrast to prostate cancer cells, no evidence was found for clonal gene somatic copy changes in stromal components or in cultured CAFs. No *TP53* mutations in stromal components were scored, while the adjacent cancer cells were positive for *TP53* mutations. Notwithstanding the presence of mitochondrial mutations in cancer cells, only one stromal specimen had a mitochondrial mutation.

Cumulatively, the studies cited above have strengthened the view that CAFs undergo a very low somatic mutation rate compared to cancer cells [45,46]. How, then, can we reconcile the discrepant results showing that somatic gene mutations have been “mapped” as a defining signature of the CAF phenotype?

Careful examination of the technical aspects of this large body of work, particularly the frequent use in the aforementioned studies of formalin-fixed paraffin embedded (FFPE) archival issue sections compared to fresh frozen specimens examined, has led to the inevitable conclusion that the reported somatic gene alterations in stroma were, in fact, experimental artefacts (reviewed in refs. [33,46]. Rummel and colleagues [47] addressed, in a meticulous paper, the controversy regarding the stability of the CAF phenotype using breast tumor specimens with a panel of microsatellite markers selected in earlier studies. These investigators evaluated genetic changes in high-quality, research-grade stromal DNA, compared them to data generated from archival specimens, and observed that high-quality DNA specimens harbored significantly fewer genetic alterations than did FFPE-treated archived tissues. It is worth noting at this point that formaldehyde, the main component of formalin, is highly reactive with DNA bases and proteins including histones, leading to DNA fragmentation degradation and artefacts in DNA sequences [48]. In the same papers [29,31], gene alterations noted in CAFs were nearly as frequent as those scored in the contiguous epithelial tumor tissue, suggesting tissue sampling contamination.

As a whole, the hypothesis that the durable phenotype of CAFs derives from mutant DNA has been severely questioned and the prevailing consensus strongly favors the view that epigenetic changes play a key role in the permanence of the CAF phenotype. This view has been adopted in a recent white paper published by the National Cancer Institute-Tumor Microenvironment Network [49]. Allowance must be made, however, for the possibility that stromal myofibroblasts—like cells exhibiting somatic mutations—derive from cancer cells via the EMT process mentioned above [10]. We address this important point later in Section 5.

In conclusion, if rare de novo genomic mutations occur in CAFs, they appear not to provide a patently selective advantage for cell growth and these inconsequential somatic gene changes would eventually qualify, at best, as passenger mutations.

## 3. Epigenetic Changes Underlie Trans-Differentiation of Stromal Fibroblasts to CAFs

We next examine the mounting evidence that epigenetic changes in CAFs—compared to wild type stromal fibroblasts—are part and parcel of their stable genomic landscape. Readers are referred to references [50,51,52,53,54] for details on epigenetic mechanisms.

### 3.1. Cancer-Associated Fibroblasts and the DNA Methylome

A large number of studies are consonant with the mechanistic view that epigenetics changes underpin the trans-differentiation of stromal fibroblasts to CAFs. Thus, Fiegl and colleagues [55] using laser capture micro-dissection of fibroblasts reported gene promoter methylation in stromal cells harvested from HER-2/neu-positive breast cancers. Hu and co-workers [56] in an extensive study used a methylation-specific digital karyotyping technique to assess the DNA methylation profile of stromal fibroblasts harvested from normal breast tissue and from primary and metastatic breast carcinomas. Findings showed distinct DNA hypomethylation in stromal fibroblasts from breast cancer in a stage-dependent manner compared to normal breast fibroblasts. In a hallmark paper, Jiang et al. [57] used methylation-sensitive SNP array analysis to profile DNA methylation in early passage cultures of stromal CAFs isolated from within-human gastric cancer specimens compared to histologically normal myofibroblasts 10 cm away from the tumor mass. The investigators noted widespread DNA hypomethylation in CAFs with sporadic focal increases in DNA methylation (e.g., the *HOX96* gene). Global DNA hypomethylation in stromal myofibroblasts was further validated by bisulfite sequencing, methylation-sensitive cytosine incorporation assay and by nuclear 5-methylcytosine (5mCyt) immunohistochemistry. Consonant with previous results, no genomic instability, such as altered chromosomal or sub-chromosomal copy number and LOH, was noted. Interestingly, PCR analysis did not show any change in the expression of the DNA methyl transferases *DNMT* 1, *DNMT*3*a* *DNMT*3*b* transcripts [50,51,52,53,54] in gastric CAFs compared to normal fibroblasts.

In a subsequent study of a *Helicobacter*-infected transgenic mouse model of gastric carcinoma [58], investigators found that stromal fibroblasts surrounding dysplastic lesions exhibited a loss of global methylation. Notably, dietary folic acid, the donor of the methyl group essential for DNMTs catalytic action [59], significantly interfered with gastric neoplasia.

Results pertaining to DNA methylation in colon CAFs are in line with the epigenetic hypothesis. An early work [60] reported DNA hypomethylation in the proteoglycan versican gene in stroma from human colon carcinomas. This is an interesting observation since the expression of full length human recombinant versican in cultured murine fibroblasts was shown to induce a myofibroblast-like phenotype [61]. It is worth mentioning that these changes required the participation of TGF-β1 signaling, a finding consistent with the recurring observation that the master cytokine regulates versican expression in the TME [62]. Mrazek and colleagues [63] assessed DNA methylation in genes from normal stromal fibroblasts compared to CAFs at the late stages of the adenoma–carcinoma sequence in CRC. Genome-wide gene expression and methylation analysis were performed using Illumina Human Expression and Illumina Human Methylation Bead Chips methodologies. The authors identified a differential methylation profile between CAFs and normal stromal fibroblasts: Compared to normal controls, 26 genes were overexpressed in CAFs as a result of promoter hypomethylation and 33 genes exhibited a lowered expression following promoter hypermethylation.

In a series of preliminary experiments designed to provide direct evidence that cancer cells instruct colon fibroblasts to acquire a CAF-like phenotype, we have assessed global DNA methylation in a human normal colon fibroblast cell line (CCD-18Co) co-cultured in a Transwell system with a human colon adenocarcinoma HT-29 line [64,65], thus mimicking a typical paracrine route of communication. Cells were cultured for 24–72 h in appropriate culture media. Following genomic DNA extraction, global DNA methylation was quantified by a microplate-based assay. Findings indicated that the paracrine cross talk of CCD-18Co cells with HT-29 cancer cells resulted in a progressive global DNA hypomethylation in colon fibroblasts. Using conditioned medium harvested from HT-29 cells, we also observed [66] that DNA hypomethylation was associated with a marked and consistent increase in mRNA and protein expression of cyclooxygenase-2 [66], an established marker of the CAF phenotype [22,67].

We recently performed [68] immunochemical staining of nuclear 5-mCyt in colon stromal fibroblasts from human biopsies taken at different stages of the CRC adenoma–carcinoma sequence. While 5-mCyt staining intensity did not differ between stromal cells in adenoma specimens and in the respective samples of adjacent normal control tissue, marked global DNA hypomethylation was consistently scored in CAFs surrounding carcinoma cells. Cumulatively, these results indicate that global DNA hypomethylation is a phenotypic characteristic of colon CAFs in CRC, the epigenetic signature of CAFs being fully “mapped” at the CRC carcinoma stage.

Genome-wide methylation microarray profiling was performed using CAFs and paired control stromal fibroblasts by Vizoso and colleagues [69] in a cohort of patients with non-small cell lung cancer (NSCLC); findings indicated extensive global DNA hypomethylation with focal gain in DNA methylation compared to normal stromal fibroblast cells.

In a recent paper, Xiao et al. [70] used combined gene methylation and expression arrays to examine human pancreatic ductal adenocarcinoma (PDAC)-derived CAFs cultured alone compared to CAFs co-cultured with PDAC cells for 24 h. Immuno-isolation methodology was used to separate fibroblasts from cancer cells. Upon direct contact with PDAC cells, DNA methylation was induced in a number of CAF genes, including the gene dubbed Suppressor of Cytokine Signaling 1 (SOCS1). Of note, SOCS1 methylation in CAFs was associated with DNMT1 overexpression. Consonant with these findings, immunohistochemistry analysis of PDAC specimens revealed diminished SOCS1 expression in cancer-associated stroma compared to normal stroma. The altered epigenetic signature of stroma supported patient-derived xenografts (PDXs) growth in mice.

Intense interest has elicited a paper by Albrengues et al. [71] describing an epigenetic switch involving the leukemia-inducible factor (LIF) which converts human head and neck cancer stromal fibroblasts to CAFs. We examine these results in detail below (Section 4.2.5).

### 3.2. Cancer-Associated Fibroblasts and Post-Translational Histone Modifications

An important route of epigenetic changes involves post-translational modifications of histone tails [50,51,52,53,54]. Surprisingly, this key avenue of epigenetic information has been somewhat neglected in the search for mechanisms underpinning the emergence of the stable CAF phenotype.

Tyan et al. [72] reported that a co-culture of breast cancer cells, with fibroblasts harvested from normal human breast tissue, induced an increase in mRNA and protein levels of ADAMS1—a metalloproteinase able to degrade ECM proteins—in the mesenchymal cells [73]. No changes in DNA methylation at the ADAMS1 promoter were found. Notably, the CAF-induced action persisted for a number of culture passages following removal of the cancer cells. Results indicated that the reduced binding of the histone methyltransferase EZH2 to the ADAMS1 promoter—and the resulting decrease in promoter methylation triggered by the repressive histone marker H3K27-me3 [50,51,52,53,54] accounted for the enhanced expression of the *ADAMS1* gene.

An interesting paper published by Zong and colleagues [74] reported that overexpression in normal prostate stroma of Hmga2, a non-histone chromatin remodeling protein involved in cell transformation [75], led to the formation of multifocal precancerous lesions in the adjacent normal prostate epithelium. This morphological perturbation was associated with increased activity of the oncogenic Wnt signaling pathway and with enhanced histone acetylation. One may tenably argue that while this observation robustly tallies with the evidence that chromatin changes in stromal cells foster the tumorigenic process in epithelial cells, *Hmga2* overexpression was forcibly induced into normal stromal cells using a lentiviral vector containing the mouse *Hmga2* coding sequence. The question, then, arises: Is the overexpression of *Hmga2* an integral part of the CAF phenotype? This important issue was addressed in a paper related to stromal fibroblasts and CRC [76]. These authors found, using immunohistochemical analysis, that the elevated expression of *HMGA2* in tumor cells was associated with tumor aggressiveness. Overexpression of human *HMGA2* was noted in a fraction of stromal fibroblasts; unexpectedly, however, it correlated with low or absent expression of *HMGA2* in tumors. Recently, Strell and co-workers [77] noted that human stromal *HMGA2* is an independent prognostic marker for ampullary adenocarcinomas and in PDAC associated with a poor prognosis in both cancer types.

A final question arises: is the stabilization of the CAF phenotype sustained by positive feedback signals inherent in the widespread autocrine mode of cell communication? Using a co-implantation breast tumor xenograft mouse model, Kojima and co-workers [21] have presented robust evidence indicating that autocrine TGF-β1 and TGF-β2 generation induced fibroblast-to-myofibroblast differentiation and conversion to CAFs. In the paper cited above, the signaling pathways up-regulated by both TGF-β1 and SDF-1 elicited the endogenous expression of the respective ligands, thereby generating a self-stimulating, bi-directional loop that acted in a positive feed-forward manner. The authors stated that “Such autostimulating signaling may fulfill the prerequisites of an epigenetic mechanism that can stably maintain a cellular phenotype” (verbatim)”.

Marks et al. [78] have published an incisive, well-balanced review focused on the epigenetic control of TME to be perused by readers for further information on this interesting issue. Other reviews have discussed genetic and epigenetic alterations in CAFs and in other TME cell populations [79,80].

**Figure 1:**
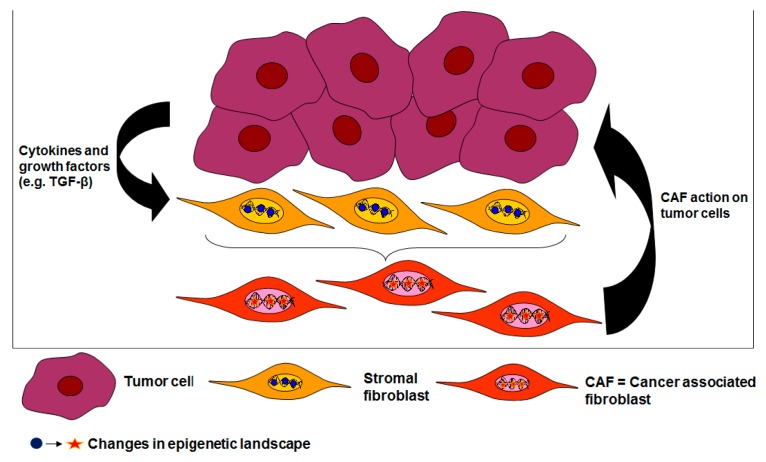
New epigenetic marks are imposed on normal stromal fibroblasts by neighboring cancer cells reshaping their epigenetic landscape. Cancer-derived cytokines such as TGF-β1 are involved in this process. The altered epigenetic signature accounts for the durable phenotype of cancer-associated fibroblasts (CAFs). Once corrupted to CAFs, stromal fibroblasts support the relentless growth and invasive program of the cancer cells. Note the vicious, self-perpetuating sequence of events that propagate and maintain the malevolent liaison between cancer cells and CAFs.

In conclusion, a vast body of evidence supports the view that epigenetic switches underpin the durability of the CAF phenotype. Figure 1 shows a schematic representation of epigenetic changes imposed by cancer cells on normal stromal fibroblasts and the subsequent vicious circle that fosters the cancer process.

## 4. The Involvement of TGF-β1 Signaling in Shaping the Epigenetic Landscape of Cancer-Associated Fibroblasts

### 4.1. A Synopsis of TGF-β1 Signaling

Before examining, in detail, the experimental evidence supporting the involvement of TGF-β1 in re-programming the epigenetic signature of stromal fibroblasts, we provide a brief description of the master cytokine as a busy purveyor of key biological information to target cells.

The TGF-β1 protein is the prototypic member of a large family of growth factors playing a crucial role in embryonic development, in adult tissue homeostasis and in cancer development [81]. Fibroblasts secrete TGF-β1 into the extracellular matrix (ECM) in the form of an inactive homodimeric polypeptide non-covalently associated with two proteins: a latency-associated peptide (LAP) that encloses the latent form of the cytokine within a straitjacket-like structure covalently bound to a large TGF-β1 binding protein (LTBP). The LAP-TGF-β1-LTBP ternary complex is subsequently secreted into the ECM [82,83,84,85].

The activation of latent TGF-β1 to the signaling ligand form entails its dissociation from LAP. Escape from the ECM cage is promoted by a variety of mechanisms, and binding of the complex to cell surface integrins is a prominent one [82,83]. This integrin class recognizes a tri-aminoacid motif (RGD) present in the pro-domain of latent TGF-β [82]. The heterodimer integrin proteins function as transmembrane protein linkers between the ECM and the actin cytoskeleton [84], transmitting contractile forces to the LAP-latent TGF-β1-LTB trio in the ECM. This mechanical pulling provokes a conformational change that liberates TGF-β1 from its inactive state. Interestingly, the activation of TGF-β1 increases the transcriptional expression of integrins in both epithelial and fibroblast cells [85,86]: thus, both the release and the availability of the potent cytokine is amplified by a feed-forward circuit.

Integrins are an integral part of a large number of ECM stromal modulators of TGF-β1 in cancer; a comprehensive and incisive review focused on this issue has been recently published [87].

Once released from the ECM cage, active TGF-β1 is ready to impart its rich informational content to target cells via autocrine, cell-to-cell contact and paracrine routes or using the exosomal pathway [88].

**Figure 2:**
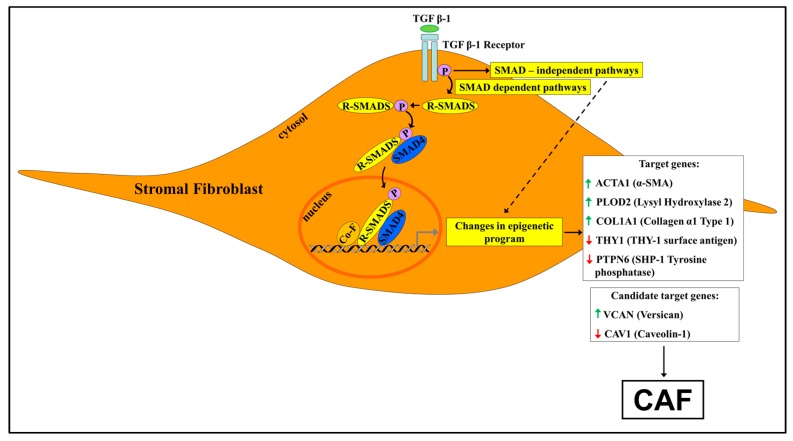
Cancer-derived TGF-β1 reshapes the epigenetic signature of normal stromal fibroblasts surrounding a growing tumor via SMAD-dependent and SMAD-independent signaling pathways. For clarity’s sake, only the SMAD-dependent pathway is shown. TGF-β1 signaling induces changes in nuclear enzymes and regulatory proteins such as DNMTs, histone-modifying enzymes and BET proteins governing the multi-tiered epigenetic program of fibroblast cells. The outcome is chromatin reprogramming and the generation in the tumor microenvironment (TME) of pro-tumorigenic CAFs bearing a stable phenotype. CAFs, in turn, secrete TGF-β1 and therefore participate in a vicious pro-tumorigenic cycle shown in Figure 1. Target candidate genes denote stromal fibroblast genes known to be regulated by TGF-β for which, in contrast to target genes, experimental evidence for TGF-β reshaping of epigenetic landscape is, to date, circumstantial. This figure is based on selected papers discussed in the review presenting evidence for TGF-β1-induced alterations in the epigenetic signature of stromal fibroblasts during trans-differentiation to CAFs in cancer (target genes) or in myofibroblasts resident in non-neoplastic fibrotic loci (candidate genes). **Green** arrow: up-regulation. **Red** arrow: down-regulation. In brackets: the coded protein. Co-F: co-factors.

At the membrane surface of a responding cell, TGF-β1 binding activates a heteromeric complex of transmembrane receptors referred to as type I and II, each equipped with an intracellular serine–threonine kinase domain. The close encounter of the cytokine with the extracellular domain of receptor type II triggers phosphorylation and activation of type I receptor (Figure 2) [89]. In turn, receptor type I phosphorylates, on their carboxy terminal, end intracellular cytosolic transducer proteins referred to as SMADs. These regulatory SMADs (R-SMADs) are represented in the TGF-β family by R-SMAD2 and R-SMAD3. Once phosphorylated, the R-SMAD effectors complex in the cytosol with a common SMAD4, and the heteromeric assembly of R-SMADs and SMAD4 translocates to the nucleus to interact with a specific DNA SMAD binding element (SBE), ultimately acting as transcription factors that regulate the expression of a vast array of genes [89,90]. Importantly, a single SBE is not sufficient to bind the SMAD complex; moreover, the affinity of SMADs for DNA is feeble. Consequently, nuclear SMADs recruit additional transcription or adaptor factors, including chromatin modifiers to execute their extensive program of control of gene expression [89,90]. These multi-subunit transcriptional protein complexes play a vast number of different biological roles, acting either as gene activators or repressors [89,90].

In addition to the direct canonical, SMAD-dependent pathway outlined above, the TGFβ-I receptor, once phosphorylated and activated, engages non-SMAD-mediated signaling pathways—such as MAPK and PI3K pathways—and these alternative routes of TGF-β1 action are collectively referred to as non-canonical or SMAD-independent pathways [91].

Once free, TGF-β1 signaling generates combinatorial signals and context-, time- and location-dependent biological responses in different cells or even in a single cell at different stages of development and function. Many nuclear associates of SMADs are tissue-specific transcription factors and consequently they impose a context-dependent gene regulation, explaining, at least partly, the bewildering contextual reply of cells to TGF-β1 signaling inputs. Consequently, at any moment, the response of a cell to TGF-β1 challenge is the permutation and summation of signaling networks finely tuned to its homeostatic demand [89,90,91,92,93]. Dysregulation or misuse of this multi-level regulatory system of control has the potential to trigger cellular and tissue functional havoc.

Among the principal regulatory molecules antagonizing TGF-β1 activity are the inhibitory SMADs (I-SMADs) which include I-SMAD6 and I-SMAD7 [94]. These inhibitory proteins act at several levels of TGF-β/SMAD signaling. It is worth noting that an early paper [95] reported that TGF-β1 enhances the expression of SMAD7 mRNA, indicating a TGF-β1-dependent negative feedback loop restraining the activity of the cytokine.

Additional proteins negatively regulate TGF-β1/SMAD signaling at the post-transcription level. These include the extensively studied SMAD ubiquitin regulatory factors denoted by the whimsical abbreviation of SMURFs. SMURFs are E3 ubiquitin–protein ligases that control by poly-ubiquitination the stability and density of nuclear SMADs [96]. Notably, TGF-β1 was shown to target SMAD2 for degradation by the proteasomal machinery [97].

The reader interested in gaining more information on the intricate network of cues and signals finely tuning TGF-β1 signaling is directed to a recent incisive and comprehensive review [98].

A question comes to the fore at this point: Are regulatory mechanisms that govern TGF-β1-ordered signaling impaired in CAFs? Surprisingly and to the best of our knowledge, this important issue appears not to have been explored and in view of the commonality of biological behavior and phenotypes between myofibroblasts of different origins [99], we orient our attention to the TGF-β1/SMAD signaling pathway in myofibroblasts from non-neoplastic chronic diseases. Thus, in dermal myofibroblasts from idiopathic fibrotic scleroderma, a disease characterized by copious fibrosis, SMAD7-SMURFs-mediated negative regulation of TGF-β1 signaling appears to be impaired, allowing sustained autocrine activity of the cytokine [100]. These results make the question raised above tenable and deserving of close scrutiny.

### 4.2. TGF-β1 Signaling and the Epigenetic Signature of Cancer-Associated Fibroblasts

We have reviewed previously the robust evidence that epigenetic changes define the signature and the stability of the CAF phenotype. A key question comes to the fore: which cues and signals reshape the epigenetic landscape of stromal fibroblasts?

The pervasive involvement of TGF-β1 in the induction of fibroblast-to-myofibroblast trans-differentiation and in the shaping of the CAF phenotype is amply documented [15,16,17,18,19,101,102,103]. We turn now to discuss, in detail, the substantial evidence—briefly mentioned in [101], showing that myofibroblast differentiation provoked by TGF-β1 is associated with epigenetic changes. To this aim, we present below a selected panel of target genes coding for CAF proteins for which robust experimental evidence for epigenetic regulation by TGF-β1 is available. Candidate target genes were also selected on the basis of circumstantial evidence that remains to be validated.

#### 4.2.1. The *ACTA2* Gene

A number of studies have unequivocally shown that TGF-β1 up-regulates in normal fibroblasts the expression of the *ACTA2* gene coding for the α-smooth muscle actin (α-SMA) protein [15,16,17,18]. This cytoskeletal protein—widely used as a CAF marker—is involved in the reorganization of the actin cytoskeleton facilitating the stiffness of collagen fibers, a hallmark of the stromal myofibroblast phenotype in TME [104,105].

A carefully designed study by Hu et al. [106], focused on the a-SMA protein, demonstrated that the differentiation of rat lung myofibroblasts in vitro is epigenetically induced by DNA methylation. Thus, the inhibition of activity or knockdown of DNMTs expression by isoform-specific siRNAs caused a significant induction of α-SMA mRNA levels, showing that DNA hypomethylation was associated with increased expression of the *ACTA2* gene. In contrast, induced overexpression of DNMTs induced ACTA2 promoter hypermethylation and blunted the expression of the *ACTA2* gene. The investigators confirmed previous studies showing that TGF-β1 up-regulates α-SMA expression in fibroblasts in vitro and in vivo [15,16,17,18]. Notably, the TGF-β1-mediated action was enhanced or suppressed by knockout or overexpression of DNMTs, respectively. Real time PCR analysis showed that TGF-β1 on its own significantly inhibited mRNA expression of DNMT1 and DNMT3α. Interestingly, these investigators have previously shown that, in rat lung fibroblasts, SMAD3 binds directly to promoter regions of the *ACTA2* gene containing SBE elements (see above) and this action was associated with the increased production of the α-SMA protein [107].

Predictably, the histone-based epigenetic arm is also exploited by TGFβ-1 in the regulation of *ACTA2* gene expression. Thus, it was shown [108] that histone de-acetylation catalyzed by HDAC4 [50,51,52,53,54] is required for TGFβ-1 to enhance α-SMA expression and to induce the differentiation of primary human skin fibroblasts to myofibroblasts. These observations are consistent with results showing that histone de-acetylase inhibitors abrogate TGFβ-1-induced myofibroblast differentiation and α-SMA induction [109]. Since Histone HDACs activity is associated with transcriptional repression [50,51,52,53,54], it was suggested [108] that the HDAC de-acetylating action might be necessary to blunt the expression of a negative repressor of TGF-β1 signaling. We propose SMAD7 as a likely candidate since de-acetylation of SMAD7 reduces the stability of SMAD7 and promotes its proteasomal demise [94].

#### 4.2.2. The *PLOD2* Gene

We now turn to genes coding for collagen precursors and the mature collagen as additional targets for the epigenetic changes induced by TGF-β1 in stromal fibroblasts. For clarity’ sake, a few introductory sentences pertaining to collagen biosynthesis and function are deemed of importance.

The ECM is composed of collagens, proteoglycans and a large number of different multi-adhesive proteins. Collagen—mostly produced by fibroblasts—is the most abundant non-cellular scaffolding protein in the TME, and its increased production is associated with tumor development and progression [110,111]. Abundant collagen synthesis and secretion is, as for the α-SMA protein, a defining hallmark of CAFs and of the desmoplastic stroma surrounding cancer cells [110,111].

The biosynthesis of mature collagen commences inside the fibroblast cell and terminates in the ECM [112,113]. In this multi-step cascade of biochemical events, a central role is occupied by lysyl hydroxylases (LHXs), intracellular enzymes that hydroxylase lysine residues of collagen α-chains, playing a key role in collagen processing and cross-linking [114]. Upon secretion into the ECM, pro-collagen molecules undergo removal of the amino and carboxyl terminal, self-assembly and cross-linking of fibrils to become the mature protein [112,113]. Like other fibrillary collagen isoforms, type I collagen is present in the ECM as a triple helix composed of two α1(I) chains and one α2(I) chain transcribed from *COLIA1* and *COLIA2* genes respectively and type II collagen composed of three α1(I) chains [112,113]. The increased synthesis and deposition of collagen in the TME leads to enhanced tissue stiffness, a potent stimulus for myofibroblast differentiation and an apt reminder that cells translate mechanical forces into clear biological signals [115].

Studies have demonstrated that TGF-β1 regulates key intra-and-extra cellular events leading to the synthesis and organization of collagen fibrils. Thus, an interesting paper [116] has shown that in human adult skin fibroblasts, TGF-β1 acting via SMAD3 and SP1—the latter a component of the multi-subunit protein assembly that accompanies nuclear R-SMADs (see above)—up-regulated the expression of *PLOD2*, the gene coding for LHX2. While the TGF-β1 action is compatible with a direct, non-epigenetic transcriptional control of the *PLOD2* gene [117], it is to be noted that the action of SMAD3/SP1 was associated with a rise of acetylated histones H3 and H4 at the *PLOD2* gene promoter site. This is a covalent modification of histone tails associated with a loose chromatin configuration leading to a transcriptional response [50,51,52,53,54]. Moreover, examination of histone methylation marks at the PLDO2 promoter site showed an increase in the level of histone H3K79me3, a histone mark promoting transcription and a decrease in the level of the repressive histone mark H4K20me3 [50,51,52,53,54], indicating that TGF-β1 action was closely correlated with the “calling” of specific histone modifying enzymes to the PLDO2 promoter site resulting in increased synthesis of the LHX2 protein; it is noteworthy that DNA methylation was not affected, notwithstanding that there is an abundance of CpG islands at the PLOD2 gene promoter site.

#### 4.2.3. The *COL1A1* Gene

In a recent study, Pan et al. [118] showed that, in isolated rat cardiac fibroblasts, TGF-β1 induced up-regulation of COL1A1 mRNA and protein expression resulted from DNA hypomethylation in the COL1A1 promoter; site-specific DNA hypomethylation and global DNA hypomethylation were caused by down-regulation of the expression of *DNMT1* and *DNMT3* genes.

#### 4.2.4. The *Thy1/CD90* Gene

Not all gene targets of TGFβ1-driven epigenetic changes in fibroblasts are involved in coding for proteins that are essential for the synthesis and contractility of collagen fibers and in the generation of the desmosplastic reaction. A salient case in point is the *Thy-1/CD90* gene coding for membrane bound thymocyte differentiation antigen, which, notably, suppresses fibroblast-to-myofibroblast trans-differentiation [119]. Neveu and colleagues [120] have shown that R-SMADs epigenetically blunt Thy-1 gene expression in primary mouse lung fibroblasts by upregulating the expression of DNMTs, thus inducing Thy-1 promoter hypermethylation. TGF-β1 silencing of the THY-1 gene was associated with fibroblast-to-myofibroblast trans-differentiation as evidenced by the up-regulation of the α-SMA protein expression and by the induction of the *COL1A1* gene. 

#### 4.2.5. The *PTPN6* Gene

In an interesting paper, mentioned previously, Albrengues and collegues [71] reported that an epigenetic switch in the stromal fibroblast *PTPN6* gene drives the conversion of fibroblast to pro-invasive CAFs. The *PTPN6* gene codes for the non-receptor tyrosine phosphatase SHP-1 which acts by dephosphorylating JAK kinases and STAT3 and thereby negatively controls the potentially oncogenic STAT3 pathway. In order to understand the cascade of biochemical events leading to an epigenetic change in the human stromal fibroblast *PTPN6* gene and the participation of TGF-β1 in this complex molecular process, it is necessary to peruse an earlier paper by Albrengues et al. [121]. These investigators reported that a pulse of TGF-β1 established a stable pro-invasive fibroblast activation state by inducing the production of the pro-inflammatory LIF; consequently, the mechanistic routes of the TGF-β1-LIF duo action were addressed. In brief, findings showed that LIFs constitutively activated the JAK/STAT3 signaling pathway in fibroblasts and this action required STAT3 acetylation by HAT p300. Acetylated STAT3 provoked, via up-regulation of DNMT3b hyper-methylation of the promoter site of the PTPN6/SHP-1, a gene coding for a SHP-1 phosphatase that normally blunts oncogenic JAK/STAT signaling. Silencing of the *SHP-1* gene led to the constitutive phosphorylation of JAK-1 driving the conversion of fibroblasts to CAFs. Notably, the epigenetic action of TGF-β1 required the mediation of LIF, a requirement met since TGF-β1 is a LIF inducer [71].

#### 4.2.6. The Bet Proteins

The final outcome of global and site-specific histone acetylation is not only determined by the combined activities of HAT and HDAC but also includes the participation of the Bromodomain and extra terminal domain BET proteins [51,52,53,54]. The BET protein family possesses a 110 amino acid domain, referred to as bromodomain, a modular domain frequently found in mammalian HATs, that recognizes acetylated lysine located in the protruding histone tails [122].

Recent work indicates cross-talk in CAFs between TGF-β1 and the BET histone “reader” proteins. Thus, Yamamoto and colleagues [123], using patient-derived xenografts (PDXs) of human PDAC, recently showed that in mice treated with JQ1, a BET inhibitor, tumor growth rates and weights were markedly reduced along with a significant decrease in the desmoplastic stroma. Importantly, exposure of human PDAC primary CAFs to JQ1 resulted in a marked alteration of their secretome and in the blunting of CAF markers. Using the CHIP-qPCR assay in exploring further the action of BETs on the biology of CAFs, the investigators noted that TGF-β1 significantly increased BRD4 recruitment to the IL6 and COL1α1 promoter sites. This epigenetic action of TGF-β1 exerted on CAFs induced an enrichment of pol II RNA polymerase at the transcription start site of the responsive genes; of note, all the TGF-β1 induced-changes were abolished by prior exposure of CAFs to the BET inhibitor. In line with these findings, investigators have recently reported [124] that the bromodomain-containing Protein 7 acts as transcription co-activator for SMADs by forming a complex with SMADs3/4 and by simultaneously binding to acetylated histones and to p300 to foster SMAD transcriptional action. Notwithstanding that epithelial cell lines were used in this work, we surmise that similar nuclear mechanisms involving cross-talk between TGF-β1 signaling and BETs are also functional in fibroblast cells.

#### 4.2.7. The *CAV-1* Gene

CAV proteins are an erased integral part of caveolae, small, bulb-shaped plasma invaginations rich in cholesterol and lipids involved in a vast array of physiological functions, the main one being the protection provided to cells against mechanical stresses [125,126].

Sanders et al. recently showed [127], in fibroblasts from human patients with idiopathic pulmonary fibrosis and in mouse lung fibroblasts after bleomycin injury, that TGF-β1 exerts an inhibitory action on *CAV-1* expression by lowering the binding of the transcription-enhancing histone mark H3K4M3 to the promoter site of *CAV-1*. Although the work cited above did not include CAFs, it is noteworthy that the suppression of CAV-1 protein levels in normal control lung fibroblasts treated with TGF-β1 was also associated with decreased global levels of transcription-enhancing H3K4Me3 histone relative to total H3 and with a decreased association of the histone with the *CAV-1* promoter site. These findings, the evidence of an inhibitory action of TGF-β1 on *CAV-1* expression in CAFs [128], and the commonality between the phenotype of myofibroblasts from neoplastic and non-neoplastic diseases [99], makes it highly probable that the suppressive action of TGF-β1 on CAV-1 expression in CAFs is also brought about via modification of the histone H3K4Me3 mark at the promoter site of the *CAV-1* gene. We have therefore included the *CAV-1* gene in the panel of candidate genes epigenetically modified by TGF-β1.

#### 4.2.8. The *VCAN* Gene

We have previously mentioned versican in the context of the DNA methylome and the epigenetic landscape of stromal CAFs (Section 3). Briefly, early findings [60] indicated DNA demethylation in stromal cells surrounding human colon carcinomas. Taking into account the robust evidence showing that TGF-β up-regulates the *VCAN* gene in CAF cells [62], we consider a tenable option to include the *VCAN* in the list of genes epigenetically modified by the cytokine during the fibroblast-to-myofibroblast differentiation.

#### 4.2.9. TGF-β Signaling and Metabolic Reprogramming of Cancer-Associated Fibroblasts

Recently, it has been reported [129] that downregulation of isocitrate dehydrogenase 3α (IDH3α) expression by TGF-β (or PDGF) signaling is a pivotal event in metabolic reprogramming of CAFs triggering a switch from oxidative phosphorylation to aerobic glycolysis in these cells, a metabolic change strongly favoring tumor growth; of note, the decreased enzyme expression accompanied fibroblast-to-myofibroblast transition. One may plausibly ask whether IDH3α down-regulation involves TGF-β-mediated changes in epigenetic events.

Summing up, a vast body of data show that TGF-β1 is able to alter the epigenetic signature of stromal fibroblasts via the regulation of DNA methylome induced by changes in the expression of DMNTs and in the biological behavior of BET proteins, and via alterations of histone posttranslational modifications, all nuclear events ultimately leading to chromatin reprogramming and to differential gene expression in the myofibroblast cell.

Figure 2 depicts, in a simplified way, genes that were shown—or are strong candidates to be—epigenetically modified by TGF-β1 during the fibroblast-to-myofibroblast differentiation in the TEM.

## 5. TGF-β1 Signaling, Cancer-Associated Fibroblasts and Neoplasia: Colon Cancer as a Paradigm for the Human Disease

In order to progress further in the transformation pathway and to acquire a selective growth advantage, the cancer cell must evade the potent cytostatic action of TGF-β1 which operates in a normal cell and at the early stages of the neoplastic process [81,92]. Accordingly, malignant clones with disabling mutations within coding sequences of TGF-β1RII or in core components of the TGF-β1 signaling pathway, such as the promiscuous SMAD4, are frequently found in patients with sporadic CRC [130,131,132,133]. The loss-of-function mutations in TGFβ-1 signaling components in CRC are fully expressed at the transition of the adenoma-to-carcinoma stage [130]. It is noteworthy that CRC cells bearing mutant SMADs, but with a functional receptor TGF-β, are able to activate non-SMAD signaling pathways upon TGF-β1 binding (Figure 2). Notably, CRC cells devoid of TGFβ signaling are able to produce TGF-β and one may ask what is the molecular logic behind the continual synthesis of a ligand in the absence of the recipient receptor.

The answer shows how opportunistic the tumor cell can be: colon cancer cells with a disabled TGF-β1 pathway but un-impaired synthesis of the cytokine exploit the “wild type” TGF-β1/SMAD signaling pathway fully operative in CAFs to elicit pro-tumorigenic signals which, in turn, impinge back on them, providing a vicious loop that ultimately sustains their relentless oncogenic agenda (Figure 1).

A salient example of this self-perpetuating sequence of events was shown by Calon and colleagues [134] who observed that colon carcinoma cell lines with a non-functional, silent TGF-β pathway drive metastasis using their un-impaired capacity to synthesize TGF-β. Notably, cancer-derived TGF-β acting on TGF-βR of colon CAFs enhanced the secretion of cytokine LIF which, in turn, promoted the survival and invasiveness of CRC cells by up-regulating the production of the oncogenic transcription factor STAT3. On the basis of these observations, one may tenably argue that TGF-β1 is not a double-edged, molecular Jekyll and Hyde of cancer [135] which, anti-mitotic and pro-apoptotic in early neoplasia, morphs into a potent cancer driver at later stages of tumorigenesis; what happens in molecular terms is that the cytokine is used via the intermediacy of CAFs by the devious cancer cells to foster their growth and invasive program.

These studies provide a logical explanation of an apparent puzzling observation: Notwithstanding that a relatively large number of CRC have mutational inactivation of the TGF-β/SMAD pathway, a high level of TGF-β in tumors correlates with poor diagnosis and disease relapse [136,137]. We may note here that mutational inactivation of core components of the TGF-β signaling cascade has been observed in other types of sporadic advanced solid tumors, such as hepatocarcinomas and pancreatic cancer [138,139,140]. In contrast, a number of solid tumors, including breast, glioblastomas and medulloblastomas possess a fully functional TGF-β/SMAD signaling pathway [81,92]. In these tumors and in colon tumors not bearing mutations in the TGF-β1 signaling pathway, the cancer cells co-opt into their own service several TGF-β-initiated pathways that are not oncogenic in a normal cellular context, again turning TGF-β into an unwitting pro-tumorigenic agent [81,92].

Based on the assessment of global gene profiling, an international consortium has recently identified [141] four consensus molecular CRC subtypes (CMSs), and a common observation has been that the stem cell-like mesenchymal CRC subtype 4 (CMS4) characterizes a particular class of highly aggressive colon tumors portending a worse diagnosis. Notably, CRC in this patient group exhibits high TGF-β activation, stromal invasion and angiogenesis. These molecular classification systems have further improved the stratification of patients with CRC, providing an important step towards a better treatment and personalized medicine [142].

In a study focused on the molecular stratification and prognosis of CRC based on global gene expression profiles of micro-dissected and FACS analyzed cells, Calon and colleagues [143] showed that in aggressive CRC subtypes characterized by resistance to treatment and unfavorable prognosis, erased regulated genes were of stromal origin—predominantly from CAFs—rather than from epithelial cell components. These CRC subtypes were characterized by loss of TGF-β signaling in cancer cells but un-impaired TGF-β production. Notably, CAF enhanced the frequency of tumor-initiation cells and this promoting action was fostered by cancer-derived TGF-β. Patient-derived organoids and PDXs showed that administration of LY2115729, a small molecule inhibitor acting on TGF-βRI kinase resulted in blunting the progression of the disease.Predictably, interfering with TGF-βR1 biological action was restricted to stromal cells since the colon tumor cells examined in this study bore a mutant, disabled TGF-βR.

The involvement of CAF-derived TGF-β1 signaling in colon cancer was previously shown by the extensive work of Hawinkels et al. [137]. These investigators presented findings showing that in human CRC, active TGF-β1 levels in colon tissue homogenates were increased only at the carcinoma stage. Interestingly, the CRC stage—at which active TGFβ was manifestly expressed in neoplastic colonocytes—coincides with the frank global DNA hypomethylation of human CRC CAFs observed in our study [68].

A paper by Isella et al. [144] presented robust evidence for the stromal contribution to the CRC transcriptome. CRC expression data from patient-derived cancer tissues were obtained by transplantation of tumor tissue from PDXs into immuno-deficient mice. Since human stromal cells, erased from PDXs, were substituted by stromal mouse cells, it was possible to distinguish between the expression of murine and human transcripts in cancer epithelial cells and in stromal cells using species-specific microarray platforms. The results, amply confirmed by immunohistochemical analysis, showed that transcripts were mostly stromal CAFs in origin. Collectively, the above studies based on the interrogation of CRC transcriptomes indicate that gene signatures of predictive power associated with poor CRC diagnosis and therapy resistance derive mostly from the stroma; these findings also show the importance of TGF-β signaling in determining a poor prognosis at advanced stages of the disease. Indeed, TGF-β1 target genes involved in collagen synthesis (Section 4.2) have been found to be associated with poor prognosis, and their expression has been used to stratify patients with advanced CRC or other types of cancer [145,146,147]. Importantly, a recent work by Li et al. [148] using single cell RNA and an algorithm that provided clustering precision in assessing single cell transcriptomes reported epithelial cell subgroups with different survival probability in the subtype CRCs described above, and two distinct cell populations in human CAFs, reinforcing the concept of heterogeneity of both tumor cells and CAF populations [9]. Functional heterogeneity of CAFs in colon cancer was previously shown by Herrera and colleagues [149].

We have mentioned earlier [10] that EMT represents a phenotypic switch of epithelial cancer cells to a mesenchymal phenotype promoting their migration and invasiveness. In this context, the appealing view has been advanced that EMT underpins the highly malignant aggressive nature exhibited by the CRC GMS4 subtype. However, the studies cited above regarding the origin of the transcriptome in CRC cells challenge the view that colon tumors with stem cell-like biology derive from widespread EMT. The presence of EMT-associated key genes such as SNAIl1, Twist and ZEB1 in stromal cells in colon tumors could signify that a subset of tumor cells undergo EMT, particularly at the tumor invasive front. Congruent with this view, gene expression profiles of budding cells in CRC [150] assessed by RNA-sequencing assay—compared with cells resident in the tumor bulk—indicated that cells at the tumor edge express an EMT-like phenotype closely matching the CMS4 subtype. It is worth noting that bulk cells in the same tumor were negative for *EMT* gene markers and expressed the epithelial (CMS2) phenotype.

Once more, these results show the astute and economical policy governing the cancer cell society: instead of implementing EMT in every cell, only cells at the tumor front in communication with the stroma and with an urgent need of a migratory and invasive mesenchymal phenotype activate the EMT swerve.

The reader who is interested in additional details pertaining to the close functional relationship between CAFs and TGF-β1, and the pervasive influence of the cytokine to foster CRC is referred to recent reviews [1,103].

## 6. Concluding Remarks

In this review, we have focused our attention on the epigenetic instructions of TGF-β1 in the shaping of the CAF phenotype. However, it is important to note that the master cytokine also induces tumor angiogenesis and suppresses both the innate and adaptive immune system in TME, all actions conducive to the development of cell transformation [101,140]. Moreover, TGF-β1 signals to other TME components such as endothelial and inflammatory cells. In turn, each of these cell types of different lineages interact with tumor cells, with non-transformed cell populations-including CAFs-and with the non-cellular matrix; this multi-componential network generates the heterogeneity of the TME and the cancer ecosystem [1,151,152]. Finally, notwithstanding the undeniable evidence that TGF-β1 is a prime mover imposing, via epigenetic switches, a durable phenotype on CAFs, other TME-derived cues and signals are involved in epigenetic changes and in chromatin remodeling [78,79,80].

While the investigation of therapeutic response and resistance was previously centered on tumor cell-autonomous mechanisms, nowadays the translation to the clinic of discoveries pertaining to the stroma mechanistic impact on cancer development and the targeting of tumor stromal components in cancer treatment are under intense investigation [153,154,155,156,157,158].

As noted previously, compared to cancer cells, CAFs are genetically stable without the complication of phenotypic drifts and thereby likely to be less prone to the emergence of intrinsic therapeutic resistance frequently encountered in tumor cells; this phenotypic stability, a main issue of this review, makes them attractive targets for cancer treatment. Accordingly, in the ongoing stroma-centric therapeutic approach of neoplasia, much interest is focused on CAFs as putative target cells [159,160,161,162,163].

The evidence that epigenetic modifications are malleable, plastic and potentially reversible, suggests a tenable opportunity for the re-programming of CAFs to normal fibroblasts using drugs targeting the CAF epigenome. The translation of our knowledge of the epigenomic profile of CAFs into a therapeutic approach has been incisively discussed by B. Tycko and his group with reference to DNA methylation [163]. These investigators proposed that the low expression of DMNTs in CAFs might make CAFs prone to the hypomethylation inducing-drugs, such as 5-azacytidine-2′-deoxyctidine (DAC), a potent inhibitor of DNA synthesis widely used in cancer treatment [164], thus provoking a “hypomethylation crisis” in already extensively demethylated cells.

The “hypomethylation crisis” therapeutic approach mentioned above was recently tested [165] in an aggressive mouse model of stromal-rich PDCA which exhibited global DNA hypomethylation of CAFs and of tumor epithelial cells. DAC administration significantly impeded PDAC progression: Remaining tumors showed regions of sarcomatoid changes with a marked loss of CAFs.

One may tenably argue that by interfering with stromal DNMT expression, drugs may also deprive TGF-β1 of a key ‘substrate’ needed for regulation of the epigenetic signature of CAFs.

Intense interest is focused on the BET bromodomain as a target of epigenetic therapy in cancer [166,167,168]. The response to specific inhibitors of BET proteins in CAFs and of other stromal TME cell components is therefore of prime interest and deserves close scrutiny. A similar therapeutic approach should be taken in the context of histone-modifying enzymes.

The TGF-β1 signaling pathway is increasingly considered an important therapeutic target not only because of its role in cancer cells but also because of its capacity to instruct a pro-tumorigenic program in tumor stromal cells. A wide number of therapeutic agents that interfere with the biological action of TGF-β in cancer are currently available and a number of clinical trials are in an advanced state [2,140,169,170]. Extensive discussion on the promises and limitations of these anti-TGF-β treatment approaches in CRC and in other neoplastic diseases is found in recent excellent reviews [2,140,169,170].

Another exciting approach was recently taken by Bollong and co-workers [171] using in vitro studies and mouse models of human fibrotic diseases. The investigators adopted an imaging-based screening to identify an antifungal drug capable of interfering with murine and human fibroblast-to-myofibroblast differentiation. In a series of experiments, the antifungal drug itraconazone acted as an inhibitor of myofibroblasts formation in mouse and human tissues in vitro. This work led to the generation of a small molecule effective in vivo against rodent models of skin, lung and liver fibrosis. The novelty of this work is that myofibroblasts were not the actual target of drug intervention since the research attention was focused on upstream cellular and molecular processes leading to their formation.

Notwithstanding the importance of different cancer treatments focused on the stroma outlined above, we and others [172,173] hold the opinion that, in contrast to the inhibition or ablation of specific stromal components, remodeling and reprogramming cell populations is obviously the most natural route for modifying TME. The effectiveness of this view has been shown in recent papers [174,175].

As discussed in this review, therapeutic targeting of CAFs and of other cell populations in the TME is based on a strong rationale; however, we believe that a therapeutic attack aimed at both cancer cell and the rogue stromal fibroblasts will be more effective in cancer therapy.

In this review, our attention has been focused on changes in the epigenetic signature of CAFs. A final key final question lingers: As part of the vicious circle shown in Figure 1, are CAFs on their own able to modify the epigenome of cancer cells? In line with our query, a recent paper of Sherman et al. [176] shows that stroma-derived signals provoke histone acetylation in the epigenome of pancreatic cancer cells.

Lastly, due apologies. Space constraints have made it impossible to cover the cross-talk between TGF-β1 and microRNAs in shaping the epigenetic signature of CAFs. The interested reader is directed to recent reviews for details on this compelling issue [177]. The authors also apologize for the omission of papers highly relevant to issues covered in this review.

## 7. Few (Out of Many) Outstanding Questions

1. How precisely does the ECM evolve prior its final cellular and non-cellular TME aspect? What are the intermediate stages?

2. What are the molecular mechanisms that make stromal fibroblasts protective in some kinds of cancers?

3. Are there markers unique to the CAF phenotype which are not shared by naïve fibroblasts in normal tissues or expressed in cancer cells?

4. What is the true impact of EMT in cancer cells in providing stromal CAF-like populations?

5. Do fibroblasts exposed to local stimuli such as hypoxia and mechanical stress facilitate pre-neoplastic events in adjacent normal epithelial cells?

## Abbreviations

CAFs	Cancer-associated fibroblasts
TGF-β1	Transforming Growth Factor-β1
CRC	Colorectal cancer
TME	Tumor microenvironment
EMT	Epithelial–mesenchymal transition
ECM	Extracellular matrix
LOH	Loss of heterozygosity
FFPE	Formalin-fixed paraffin-embedded
5mCyt	5-methylcytosine
DNMT	DNA methyl transferase
NSCLC	Non-small cell lung cancer
PDAC	Pancreatic ductal adenocarcinoma
JAK	Janus kinase
STAT	Signal transducer and activator of transcription protein
SOCS1	Suppressor of cytokine signaling 1
LIF	Leukemia-inducing factor
LAP	Latency associated peptide
LTBP	Large TGF-β binding protein
SMAD	Suppressor of mothers against decapentaplegic
SMURF	SMAD ubiquitin regulatory factor
a-SMA	α-smooth muscle actin
LHX	Lysyl hydroxylase
HDAC	Histone deacetylase
HAT	Histone acetyl transferase
CMS	Colorectal cancer mesenchymal subtype
